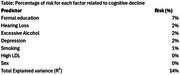# Modifiable Risk Factors and Their Potential Impact on Conversion from Normal Cognition and MCI to Dementia: Findings from a Cohort Study of Brazilian Older Adults

**DOI:** 10.1002/alz70860_105966

**Published:** 2025-12-23

**Authors:** Maria Aparecida Camargos Bicalho, Gabriela Tomé Oliveira Engelmann, Giovanna Correia Pereira Moro, Thaise Vallesca Queiroz, Marco Aurélio Romano‐Silva, Marco Túlio Cintra, Marco Aurélio Fagundes Angelo, Rafaela Teixeira de Ávila, Luiz Armando Cunha de Marco, Bernardo de Mattos Viana, Jonas Jardim de Paula

**Affiliations:** ^1^ Department of Clinical Medicine, Faculty of Medicine, Universidade Federal de Minas Gerais (UFMG), Belo Horizonte, Minas Gerais, Brazil; ^2^ Geriatrics and Gerontology Center Clinical Hospital of Universidade Federal de Minas Gerais, Belo Horizonte, Minas Gerais, Brazil; ^3^ Older Adult's Psychiatry and Psychology Extension Program (PROEPSI), School of Medicine, Universidade Federal de Minas Gerais (UFMG), Belo Horizonte, Minas Gerais, Brazil; ^4^ Cog‐Aging Research Group, Universidade Federal de Minas Gerais (UFMG), Belo Horizonte, Minas Gerais, Brazil; ^5^ Universidade Federal de Minas Gerais, Belo Horizonte, Brazil; ^6^ Jenny de Andrade Faria Institute – Outpatient Reference Center for the Elderly, Universidade Federal de Minas Gerais (UFMG), Belo Horizonte, Minas Gerais, Brazil; ^7^ Molecular Medicine Postgraduate Program, School of Medicine, Universidade Federal de Minas Gerais (UFMG), Belo Horizonte, Minas Gerais, Brazil; ^8^ Universidade Federal de Minas Gerais, Belo Horizonte, Minas Gerais, Brazil; ^9^ Undergraduate Medicine, Faculty of Medicine, Universidade Federal de Minas Gerais (UFMG), Belo Horizonte, Minas Gerais, Brazil; ^10^ Federal University of Minas Gerais, Belo Horizonte, Minas Gerais, Brazil; ^11^ Department of Psychiatry, School of Medicine, Federal University of Minas Gerais, Belo Horizonte, Minas Gerais, Brazil; ^12^ Neurotec R National Institute of Science and Technology (INCT‐Neurotec R), Faculty of Medicine, Universidade Federal de Minas Gerais (UFMG), Belo Horizonte, Minas Gerais, Brazil; ^13^ UFMG, Belo Horizonte, Minas Gerais, Brazil; ^14^ Cog‐Aging Group Research, Brazil, Belo Horizonte, Minas Gerais, Brazil; ^15^ FUMEC, Belo Horizonte, Brazil; ^16^ Molecular Medicine Postgraduate Program, Faculty of Medicine, Universidade Federal de Minas Gerais (UFMG), Belo Horizonte, Minas Gerais, Brazil; ^17^ Geriatrics and Gerontology Center Clinical Hospital of University of Minas Gerais, Belo Horizonte, Minas Gerais, Brazil; ^18^ Cog‐Aging Research Group, Belo Horizonte, Minas Gerais, Brazil; ^19^ Hospital das Clínicas da UFMG, University Hospital, Universidade Federal de Minas Gerais (UFMG), Belo Horizonte, Minas Gerais, Brazil; ^20^ Jenny de Andrade Faria Institute – Outpatient Reference Center for the Elderly, Universidade Federal de Minas Gerais (UFMG), Belo Horizonte, Minas Gerais, Brazil; ^21^ Older Adult's Psychiatry and Psychology Extension Program I Federal University of Minas Gerais, Belo Horizonte, MG, Brazil

## Abstract

**Background:**

Approximately two‐thirds of people with dementia worldwide live in LMICs. Factors such as a rapidly aging population, a high prevalence of cardiovascular risk factors, low levels of education, and social inequalities are associated with an increased number of dementia cases in these regions. Potentially modifiable risk factors (MRFs), extensively studied in HICs, may have a different impact on LMIC populations, such as Brazil. This study evaluated the impact of selected MRFs for dementia in participants from the Cog‐Aging Study, a cohort of Brazilian older adults with different cognitive backgrounds.

**Method:**

We included 851 older adults: 263 without cognitive impairment, 352 with mild cognitive impairment (MCI), and 236 with dementia. For each participant, we assessed the presence of MRFs, including hypertension, diabetes, dyslipidemia, hearing and visual impairment, smoking, alcohol intake, low educational level, low or high BMI, and depression. We compared the first diagnosis received in the study with the last one to create an outcome variable: cognitive decline. Since not all participants had complete data, we used univariate chi‐square tests to analyze individual MRFs as predictors of cognitive decline and a stepwise multivariate logistic regression model to evaluate all variables.

**Result:**

Univariate analysis revealed that dyslipidemia (*p* = 0.004), excessive alcohol consumption (*p* = 0.012), hearing loss (*p* = 0.018), and a family history of dementia (*p* = 0.002) were associated with a higher risk of cognitive decline. The regression model was significant (*p* = 0.037) and showed moderate effect sizes (Cox & Snell *R* ^2^=0.14) (Table 1). We selected the fifth of eight steps, which explained the most variance while remaining statistically significant. The predictors of cognitive decline were low educational level (OR=0.08, *p* <0.001), hearing loss (OR=1.95, *p* = 0.024), excessive alcohol consumption (OR=2.52, *p* = 0.003), depression (OR=2.00, *p* = 0.019), and smoking (OR=0.51, *p* = 0.041). The model accurately classified 75% of the sample and showed high specificity (93%) but low sensitivity (31%).

**Conclusion:**

The results of this cohort study of Brazilian older adults receiving care through the Brazilian public health system suggest that sensory deprivation, along with mental health disorders and low educational levels, are the most significant modifiable risk factors for cognitive decline and should be prioritized in public health policies.